# Fundamentals for Future Mobile-Health (mHealth): A Systematic Review of Mobile Phone and Web-Based Text Messaging in Mental Health

**DOI:** 10.2196/jmir.5066

**Published:** 2016-06-10

**Authors:** Sofian Berrouiguet, Enrique Baca-García, Sara Brandt, Michel Walter, Philippe Courtet

**Affiliations:** ^1^ Brest Medical University Hospital at Bohars, Adult Psychiatry Brest France; ^2^ Department of Psychiatry at Fundación, Jimenez Diaz Hospital Madrid Spain; ^3^ Department of Psychiatry, Icahn School of Medicine at Mount Sinai, New York, USA. New York, NY United States; ^4^ Department of Emergency Psychiatry and Post Acute Care, CHRU Montpellier, University of Montpellier, Montpellier, France. FondaMental Foundation, Créteil, France Montpellier France

**Keywords:** text messaging, cell phones, mental health, Internet, medical informatics

## Abstract

**Background:**

Mobile phone text messages (short message service, SMS) are used pervasively as a form of communication. Almost 100% of the population uses text messaging worldwide and this technology is being suggested as a promising tool in psychiatry. Text messages can be sent either from a classic mobile phone or a web-based application. Reviews are needed to better understand how text messaging can be used in mental health care and other fields of medicine.

**Objective:**

The objective of the study was to review the literature regarding the use of mobile phone text messaging in mental health care.

**Methods:**

We conducted a thorough literature review of studies involving text messaging in health care management. Searches included PubMed, PsycINFO, Cochrane, Scopus, Embase and Web of Science databases on May 25, 2015. Studies reporting the use of text messaging as a tool in managing patients with mental health disorders were included. Given the heterogeneity of studies, this review was summarized using a descriptive approach.

**Results:**

From 677 initial citations, 36 studies were included in the review. Text messaging was used in a wide range of mental health situations, notably substance abuse (31%), schizophrenia (22%), and affective disorders (17%). We identified four ways in which text messages were used: reminders (14%), information (17%), supportive messages (42%), and self-monitoring procedures (42%). Applications were sometimes combined.

**Conclusions:**

We report growing interest in text messaging since 2006. Text messages have been proposed as a health care tool in a wide spectrum of psychiatric disorders including substance abuse, schizophrenia, affective disorders, and suicide prevention. Most papers described pilot studies, while some randomized clinical trials (RCTs) were also reported. Overall, a positive attitude toward text messages was reported. RCTs reported improved treatment adherence and symptom surveillance. Other positive points included an increase in appointment attendance and in satisfaction with management and health care services. Insight into message content, preventative strategies, and innovative approaches derived from the mental health field may be applicable in other medical specialties.

## Introduction

In recent years, the general public and caregivers have increasingly adopted the use of mobile phones. According to the United Nations specialized agency for information and communication technologies, the number of mobile phone subscriptions worldwide had reached almost 7 billion by the end of 2014, corresponding to a penetration rate of 96% worldwide and 90% in developed countries [1]. Mobile health (mHealth) can be defined as the use of mobile computing and communication technologies in health care and public health [2]. mHealth has the potential to incorporate qualities often associated with more conventional health communication methods, such as personalization, tailoring, interactivity, and message repetition at a relatively low cost. Text messaging (short message service, SMS) has proven to be effective, in particular, in psychiatric care. This form of communication allows for the exchange of messages containing 160 characters or fewer between mobile phones [3]. Messages can be sent in a standardized or individualized format and are available on all mobile phones, including low-cost devices. Text message frequency (daily, weekly, etc.), text message interactivity (one-way vs. two-way), personalization (message content based on known characteristics, including patient’s condition, history, etc.) and tailoring (message frequency, interactivity and/or content matching each recipient’s characteristics) [4]. SMS text messages can also be sent from web-based platforms that allow for pre-scheduling of sending, automation, and better monitoring of reception status.

There is emerging evidence that mobile phones can play an important role in health care delivery, especially in mental health [5]. Combining Internet or mobile phone contact with traditional treatment has shown meaningful results in remote counseling [6] and monitoring support [7]. Literature reviews examining the use of mobile phones in health care have demonstrated the potential of mobile phones to support health education [8], increase access to health care [9], improve prevention and treatment strategies [10], and support public health programs [11]. Text messaging has also been used to provide appointment reminders [11], improve patient adherence with treatment [12], monitor chronic conditions [13], and provide psychological support [14]. Additionally, SMS messages are used in the prevention of communicable diseases [15] and in preventive health promotion programs [16]. Text messaging has also improved service provision to population subgroups who do not typically use health services [13]. Text messages provide the opportunity to remotely access caregivers for advice [17], and mHealth can also extend prevention strategies for caregivers; another on-going challenge of 21st century medicine [18]. Knowledge proceeding from the mental health field could also be easily transferred to other specialties, considering the transversal contribution of cellular technology in providing innovative monitoring and prevention strategies.

When taking into account the rapid expansion of mHealth applications, combined with current mental health challenges, reviews of existing applications and evidence of successful messaging-supported health care strategies are needed. Reviews of text messaging have been proposed in many fields of medicine including diabetes, weight loss, and smoking cessation [4]. To better understand and validate the use of these specific mHealth applications, we conducted a review of the use of mobile phone text messaging to promote mental health.

## Methods

### Objective

The objective of this study was to provide a thorough review of the applications of text messaging in mental health care. Two review authors independently assessed all studies retrieved against the inclusion criteria (SB and EBG). Disagreements were resolved by a third review author (PC). During the literature review process, relevant studies were categorized in a two-step approach. We first performed the review of the titles and abstracts of all publications that were identified as relevant according to the inclusion criteria. Abstracts were then categorized by the type of methodology used, health condition, applications, and purposes. The full text of all publications that were not excluded during the title and abstract review stage were checked. Publications that met all inclusion criteria comprised the final sample.

### Search Strategy

**Figure 1:**
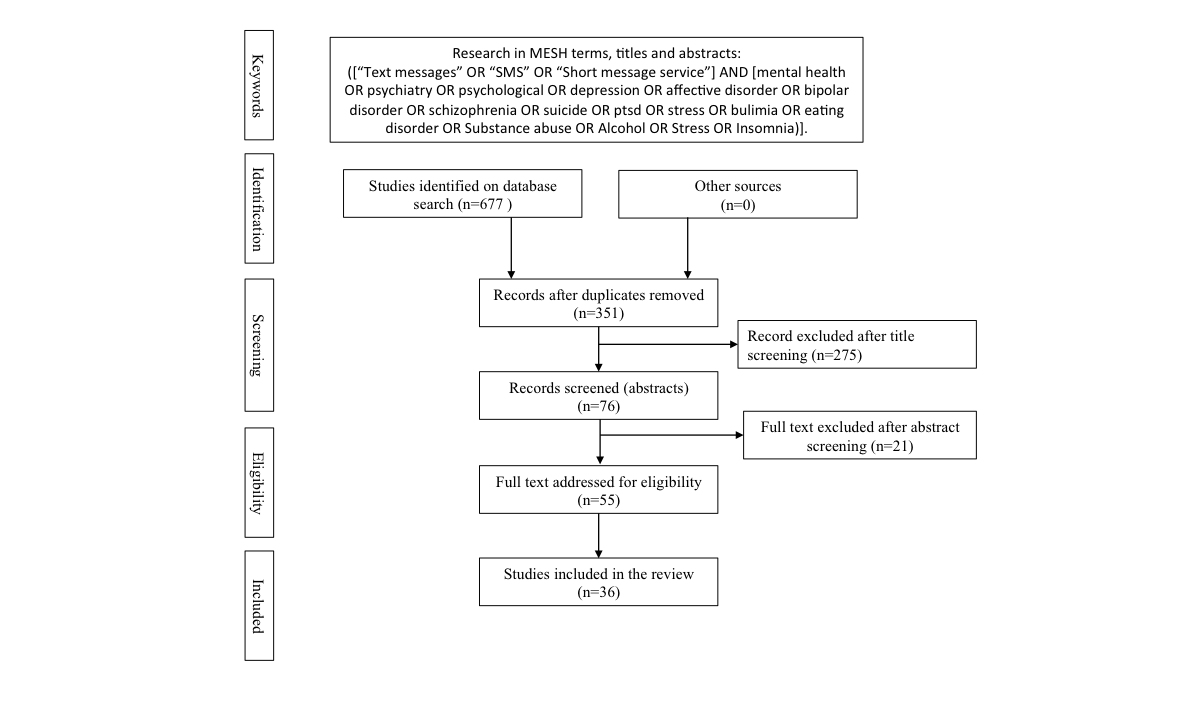
Flowchart of review process.

PubMed, PsycINFO, Cochrane, Scopus, Embase and Web of Science databases were extensively searched in May 2015. The list of keywords was created around the two domains of *mental health* and *text messaging*. A search command was constructed using “AND” and the disjunction “OR” as logical operators in Medical Subject Headings (MeSH) terms, titles, and abstracts (see Figure 1). We did not include keywords related to smoking cessation as it had already been reviewed in another article [19]. Substance abuse disorders were included in our review despite a recent study that did not take into account recent articles and pilot studies [20]. We explored titles, abstracts, and MeSH terms, and reference lists of selected studies were also checked for other potentially relevant studies.

Primary endpoints of interest were health outcomes as a result of text messaging. Technical aspects were sometimes reported, and we also considered the patients’ and caregivers’ evaluation of messaging approaches, subjective perceptions of effectiveness, institutional burden, and cost when possible. The studies included were heterogeneous in terms of design features, conditions addressed, characteristics of messaging procedure, and outcome measures. Thus, the findings are presented descriptively.

### Selection Criteria

We included randomized controlled trials (RCTs) and non-randomized studies. Study protocols were included when referring to RCTs. We included studies in which SMS text messaging was used to promote mental health, including any type of preventive or monitoring strategy. Text messages could be delivered to a patient by the caregiver or vice versa. We did not include studies assessing the patient’s general feeling about text messages (ie, surveys) for a specific medical application. We excluded studies that used mobile phone multimedia messaging service or android/OSX apps as tools for prompting.

## Results

The steps in the literature search and review process are summarized in Figure 1. The initial search retrieved 677 articles. After checking for duplicates and screening abstracts and full texts, 36 articles met the inclusion criteria. Notably, most studies (20/36, 56%) assessed the feasibility and acceptability of sending text messages to patients with a psychiatric condition or substance abuse disorder, while 44% (16/36) of the studies proposed quantitative methodologies. Ten of the 36 studies were RCTs (28%).

**Figure 2:**
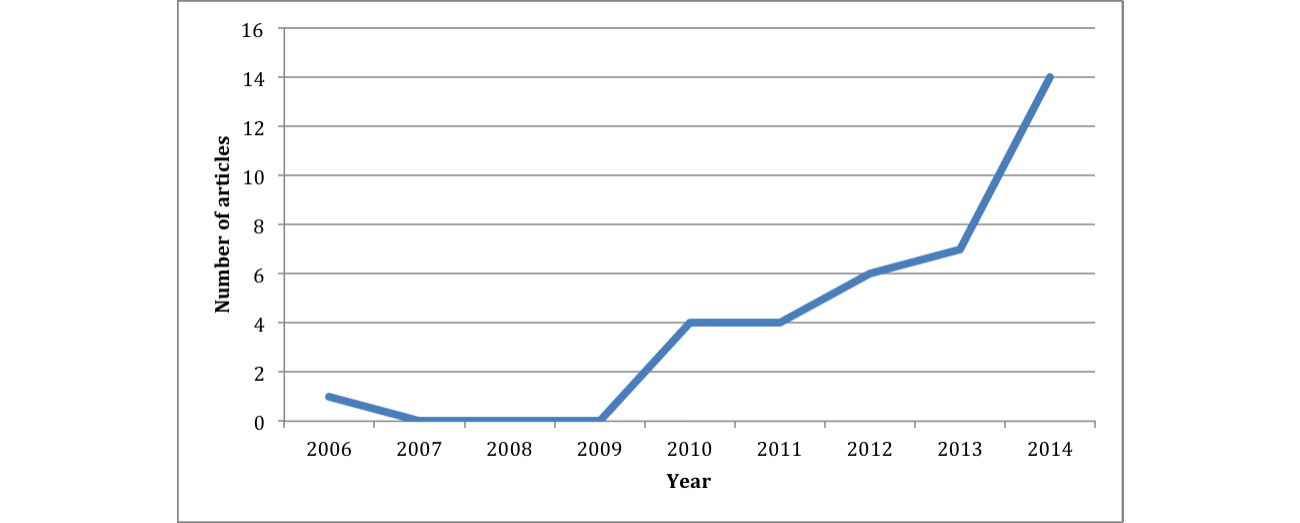
Distribution of articles over time.

We observed increased interest in text messaging, from 4 articles cited in 2010 to 14 articles cited in 2014. The largest upsurge occurred between 2013 and 2014 (see Figure 2). The majority of publications regarding text messaging were in medical journals (29/36, 81%), with the remaining 19% (7/36) being published in journals focused on medicine and telecommunication. We did not retrieve any articles published in journals focused only on technology. The geographical areas involved in this type of research were mainly Europe (16/36, 44%) and the United States (17/36, 47%).

### Health Conditions

**Figure 3:**
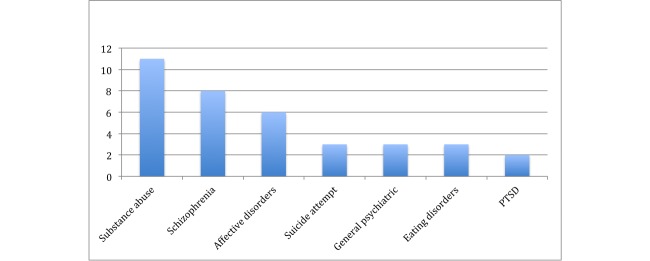
Mental health conditions addressed by text messaging.

Studies addressed a wide range of psychiatric conditions. Figure 3 shows the mental health conditions in which the use of text messaging was studied. Substance abuse conditions were most often studied (11/36, 30%), followed by schizophrenia (8/36, 22%), and affective disorders, including bipolar disorder and depression (6/36, 17%). We found only eight studies on suicidal behavior, eating disorders, and post-traumatic stress disorder (PTSD). Text messaging was used in the management of chronic conditions (27/36, 75%), reactive conditions (7/36, 19%), and preventative strategies for healthy or at-risk individuals (2/36, 6%).

### Methodology

Concerning the methodology of the studies, we identified pilot studies (20/36, 56%), followed by RCTs (10/36, 28%). Three of the RCT articles described only the study protocols with no results. Six studies used non-randomized, comparative methods. Before 2013, the samples used to study text messaging were mostly small (ie, fewer than 80 patients). Feasibility and acceptability were assessed in small sample sizes (ie, 15 to 50 patients) **.**The sample size increased significantly in 2013, with four RCTs assessing samples of more than 400 patients.

### Application

We identified four uses of text messages: reminders (5/36, 14%), information provision (6/36, 17%), supportive messages (15/36, 42%), and self-monitoring procedures (15/36, 42%). Applications were sometimes combined. Reminders aimed to improve either appointment attendance or treatment adherence. Information aimed to provide important notifications concerning available health care services or health recommendations. Supportive messages were either tailored to the patient or standardized. For self-monitoring procedures, patients had to send messages on their situation in time increments ranging from once weekly to twice daily.

### Purposes

Most studies assessed the feasibility and acceptability of text messaging for patients. Outcomes were assessed by questionnaires on satisfaction or recorded response rates. We found only one study out of 36 that concluded that messaging was not well accepted, which involved a sample of patients with eating disorders [21]. Apart from this article, the other 35 studies reported positive perception of text messaging on the primary outcomes, which were acceptability, attendance at appointments, treatment adherence, and improvement of health.

### Substance Abuse

SMS text messaging was frequently used to assess patients with issues related to substance abuse, as summarized in Table 1. A three-arm randomized trial compared self-reported alcohol use three months after emergency department visits, during which 765 young adults reported hazardous drinking [22]. Weekly text message drinking assessments were sent. The number of self-reported binge drinking days decreased from baseline to three months in the real-time feedback group compared to text message drinking assessments without feedback or a control condition. Moore et al [23] assessed the feasibility of using text messages both to survey and moderate alcohol use, and were able to determine periods of greater alcohol use (ie, weekends and celebratory events). Irvine et al [24] also used two-way text messaging as a preventative method to assess and support disadvantaged men at risk of substance abuse. Interview data indicated that text messaging was acceptable to participants, and preferred over email and web-based methods. Text messaging also proved to be accepted and effective for young people (ages 12 to 24) transitioning out of community-based substance abuse treatment programs over a 90-day period [25]. In addition to alcohol abuse, other researchers performed ecological momentary assessments (EMAs) via text messaging on daily methamphetamine use, craving levels, and the perceived usefulness of messages [26]. The odds of messages being rated as *very* or *extremely* useful were 6.6 times higher (95% CI 2.2-19.4) in the active periods than the placebo periods, signifying good acceptability for text messages.

Kuntshe and Robert [27] showed that text message reports were as accurate as reports by mail. Other teams explored perspectives concerning the appeal, acceptability, and content of text messaging in a population at risk of alcohol abuse after discharge from the emergency department [22,28], and in adolescents admitted to primary care clinics [29].

Studies also reported the use of supportive messages in substance abuse. Bedtsten et al [30] compared two methods of delivery by randomizing young students to receive automated alcohol-intervention messages, either by text messaging or by email. No difference was reported regarding satisfaction with the length and frequency of the messages, regardless of the method of delivery. Similar results were reported by Gonzales et al in young people [31]. In a longitudinal study, Haug et al [32] showed decreases in the percentages of persons with risky single-occasion drinking from baseline to follow-up assessment (75.5% vs. 67.6%, *P*<.001) in 364 students. A pilot study also examined the feasibility of a 12-week mobile-based aftercare program for youth (ages 12 to 24) transitioning out of community-based substance abuse treatment programs over a 90-day period [25]. A study protocol described the use of text messaging in heavy drinkers [33] in order to provide initial information about the feasibility and efficacy of mHealth interventions for improving treatment adherence in alcohol use disorders.

### Schizophrenia

Seven of the 36 studies focused on a diverse range of text message interventions for patients suffering from schizophrenia or related disorders, as summarized in Table 2. Two studies proposed text message reminders as a means to improve treatment adherence in patients with schizophrenia [34,35]. Montes et al [33] performed a prospective, randomized, open-label, controlled, six-month study in 56 outpatient psychiatric centers. Participants assigned to the intervention group received daily text messages on their mobile phones for three months, reminding them to take their medication consistently. The text message said, “*Please remember to take your medication*”. A significant improvement in adherence was observed among patients receiving text messages compared to the control group. Pijnenbrog et al [35] also found that patients significantly improved in keeping appointments with mental health workers when sent text message reminders, and carrying out leisure activities also increased with text message reminders.

Two studies examined text message support in outpatient management of patients with psychosis [36,37]. In the study by Ben-Zeev et al [37], the objective was to assess response rates to treatment-adherence messages. Results showed high rates of prompt responses and satisfaction rates (up to 90%) toward text message interventions. Valimaki et al [36] presented a study protocol in which the patient chose the form, content, timing, and frequency of the messages.

Symptom assessment via text messaging was proposed to patients with schizophrenia in three studies [38-40]. Ganholm et al [40] and Depp et al [38] presented two versions of the same project: Mobile Assessment and Treatment for Schizophrenia. Through this method, these studies were able to assess self-reported treatment adherence, the number of social interactions, and severity of auditory hallucinations over a 12-week period. Overall, both studies showed that text messaging for symptom monitoring is a feasible and effective form of intervention to alleviate symptoms in patients with schizophrenia. Ainsworth et al [39] compared two methods of assessing symptoms via mobile phone, and demonstrated that it was feasible using either text messages or native smartphone applications.

### Affective Disorders

Text message interventions were proposed to patients with affective disorders, including depression and mania [41-46], as summarized in Table 2. Supportive messages are used to convey positive feelings from the caregiver to patients with affective disorders. Agyapong et al [47] sent supportive twice-daily messages to a sample of 54 patients over a 3-month period. During this period, patients in the intervention group had significantly lower scores on the Beck Depression Inventory-II than control groups (8.5, standard deviation [SD] 8.0 vs. 16.7, SD 10.3, *P*=.003) [44]. Aguilera [46] also used supportive text messaging in adjunct to cognitive-behavioral therapy for depression in two English-and Spanish-native speaking populations. The findings proved the interventions were feasible and acceptable to patients and, for Spanish-speaking patients, were also related to increased feelings of being cared for.

Symptom assessment was proposed by Bopp et al [42] to assess the course of bipolar disorder symptoms through a weekly assessment with the Altman Self-Rating Mania Scale and the Quick Inventory of Depressive Symptoms-Self Report. Adherence with the procedure was high (75%) and participants more frequently reported depressive symptoms (47.7%) than manic symptoms (7%).

### Eating Disorders, Suicidal Behavior, and Post-Traumatic Stress Disorder

Text messaging has also proven to be acceptable and effective in a number of other mental disorders, such as eating disorders, suicidal behavior, and PTSD. Studies regarding text message interventions in patients with these conditions are summarized in Table 3. Three studies aimed to determine whether text message interventions might be of use in the management of eating disorders. Robinson et al [21] showed a low response rate to the self-monitoring procedure, suggesting that the intervention was only moderately well accepted by participants. However, Shapiro et al [48] showed that 87% of participants adhered to self-monitoring and reported good acceptability. Bauer et al [49] added tailored feedback to self-monitoring. In an RCT, this study reported that text messaging could support significant improvement in remission rates after discharge from inpatient treatment for bulimia nervosa. Two studies explored the feasibility of post-acute crisis text messaging outreach for suicide prevention. Weekly text messages were sent to patients after a suicide attempt. Efficacy on further suicide attempts is currently being assessed in an RCT. In both studies, messages were identical for all patients. Berrouiguet et al [50] used a web-based application in order to provide personalized and tailored messages, and to manage a larger number of patients with the end goal of reducing the rate of suicide reattempts.

Studies relating to PTSD management showed that text messaging is applicable for support of at-risk patients [51] and for monitoring acute symptoms [52]. Other messaging interventions were proposed to patients during routine follow-up, without targeting any specific disorder. These applications were essentially appointment reminders for outpatients [53,54] and preventive information for disadvantaged patients [55].

**Table 1:** Summary of text message interventions in patients with substance abuse.

Authors (year)	Country	Population (sample)	Text messages	Principal outcome	Method, duration	Result
Suffoletto (2011)	USA	Young adults from urban emergency departments (n=45)	Text messages self-monitoring	Feasibility of heavy drinking days and drinks per drinking days, assessment by text message.	Randomized comparative study	Feasible
Stoner et al (2012)	USA	Treatment-seeking heavy drinkers (expected n=105)	Medication reminders and assessment	Effectiveness	Randomized trial	To be published
Haug et al (2013)	Switzerland	Vocational school students (n=477)	Self-monitoring	To evaluate appropriateness	Longitudinal pre-post study	Study found reduced percentage of persons with risky single-occasion drinking from baseline (75.5%, 210/278) to follow-up assessment (67.6%, 188/278, *P*<.001)
Keoleian et al (2013)	USA	Methamphetamine users (n=5)	Self-monitoring	Feasibility	Randomized crossover pre-test pilot study	79% of scheduled assessment were collected.
Mason et al (2013)	USA	College students with alcohol problems (n=18)	Self-monitoring and supportive messages	Feasibility and effectiveness	Randomized trial	Text messages for alcohol abuse prevention are feasible.
Rios-Bedya et al (2013)	USA	Adolescents recruited in primary care clinics (n=29)	Ecological momentary assessment	Feasibility	Pilot study	High participation rate
Bendsten et al (2014)	Sweden	University students (n=454)	Self-monitoring and supportive messages	Satisfaction regarding text messages	Randomized trial	No difference was seen regarding satisfaction with length and frequency of messages, regardless of method of delivery.
Lucht et al (2014)	Germany	Inpatient after alcohol detoxification (n=80)	Information about telephone support, twice a week.	Controlled prospective open pilot study.	Pilot study	Feasibility and acceptability were good. Adherence was satisfactory with 57.14% of participants replying to at least 50% of prompts.
Moore et al (2014)	UK	Alcohol consumers recruited in university (n=80)	Self-monitoring	Acceptability	Randomized controlled trial	Acceptable and preferred to email conducted assessment
Rachel Gonzales et al (2014)	USA	Young participants transitioning out of substance abuse program (n=80)	Self-monitoring, supportive messages	Feasibility	Random	A significant effect of condition on primary drug use relapse outcomes over time was observed as measured by urine analysis.
Suffoletto et al (2014)	USA	Young adults discharged from emergency department (n=765)	Self-monitoring	Satisfaction towards text message or email contact	Randomized trial	Decreased number of binge drinking in web intervention group only

**Table 2:** Summary of studies using text messages in patients with schizophrenia and affective disorders.

Authors (year)	Country	Population (sample)	Text messages	Principal outcome	Method, duration	Result
Pijenborg et al (2010)	Netherlands	Patients suffering from schizophrenia with severe cognitive impairment (n=62)	Text message reminders	Improvement in functioning in daily life	Non-randomized controlled trial, 7 weeks	The overall percentage of goals achieved increased with prompting (eg, appointments). Patients enjoyed receiving the message.
Depp et al (2010)	USA	Patients with severe mental illness (n=8) in pilot study program	Text messages for EMA	Feasibility evaluation	Pilot study	Monitoring symptoms of patients suffering from severe mental illness using text messages is feasible.
Granholm et al (2012)	USA	Patient suffering from schizophrenia (n=55)	Self-monitoring	Responding rate	Pilot study, 12 weeks	Text messaging interventions are feasible and effective in patients with schizophrenia.
Maritta Välimäki et al (2012)	Finland	Patient with psychosis (protocol)	Text message support	To evaluate the impact of text messages to encourage treatment adherence and follow-up	Randomized trial, 12 months	To be published
Montes et al (2012)	Spain	Patients suffering from schizophrenia (n=254)	Daily text message reminders	Impact of text messages on adherence with antipsychotic treatment	Multicenter, randomized, open-label, controlled study, 3 months	Significant improvement in adherence
Ainsworth et al (2013)	UK	Patients with non-affective psychosis (n=24)	Symptom assessment via text messages or native smartphone application (EMA)	Compare text message based assessment strategies to native mobile texting application in terms of satisfaction	Randomized, repeated measure, crossover design, 3 weeks	A greater proportion of data points were completed with the native smartphone application.
Bebee et al (2014)	USA	Outpatients followed for schizophrenia (n=30)	Self-monitoring	To evaluate the impact of text messages on treatment adherence	Comparative study with random assignation in intervention groups, 3 months	Non-significant effect on treatment adherence
Ben-Zeev et al (2014)	USA	Patients with psychotic disorder and substance abuse (n=70)	Text message support	Feasibility and acceptability	Pilot qualitative study, 12 weeks	90% of patients found the intervention useful

**Table 3:** Summary of text messaging in outpatients with PTSD, suicide attempters, and patients with anorexia and/or bulimia.

Authors (year)	Country	Population (sample)	Text intervention	Principal outcome	Method, duration	Result
Chandra et al (2014).	India	Girls in the age range of 16-18 years from urban slums (n=40)	Information messages about health promotion	Feasibility and acceptability	Pilot qualitative study, 1 month	Mobile text messages are a feasible and culturally acceptable method for mental health promotion.
Kunigiri et al (2014).	UK	Psychiatry outpatients (n=2556)	Text message reminders	Attendance at follow-up appointment	Three-arm comparative study, text messages sent 14 days and 2 days prior to appointment	Significant increase in the attendance in text message reminder group compared to telephone
Branson CE et al (2013).	USA	Psychiatry outpatient (n=48)	Text message reminders	Text message reception rate	Pilot study exanimating technical feasibility	Patients received 88% of scheduled text messages. High patient satisfaction reported.
Price et al (2014).	USA	Patient suffering from PTSD (n=29)	Self-monitoring	Responding rate	Pilot study, 3 months	Text message described as a viable method to monitor PTSD.
Furber et al ( 2014)	Australia	Patient presenting to the Emergency Department for emotional crisis (n=68)	Supportive messages	Text message intervention acceptance rate	Non-randomized comparative prospective study (compared to historical control group, 6 months	66% of patients accepted the intervention. No significant differences in clinical outcomes between groups.
Chen et al (2011)	China	Patients discharged after suicide attempt (n=15)	Supportive messages	Feasibility and acceptability of messages to attempters after discharge	Pilot study, 1 month	Seen as feasible and acceptable to suicide attempters. Showed desire to keep receiving message.
Berrouiguet et al (2014)	France	Patients discharged after suicide attempt (n=15)	Supportive and information messages	Feasibility and acceptability	Pilot study, 12 months	Suicide attempters accepted text messages.
Berrouiguet et al (2014)	France	Patients discharged after suicide attempt (n=520)	Supportive and information messages	Suicide reattempts	Study protocol of randomized controlled study	To be published
Robinson et al (2006)	UK	Patients with bulimia nervosa (n=21)	Self-monitoring	Acceptability and feasibility	Pilot study, six months	Low participation rate and high attrition rate
Shapiro JR et al (2010)	USA	Patients with bulimia nervosa (n=31)	Self-monitoring	Participation rate	Pilot study, six months	87% of participants adhered to self-monitoring.
Lucht et al (2014)	Germany	Patient with bulimia nervosa (n=165)	Self-monitoring	Impact of text messaging on remission rate after 8 months	Randomized controlled trial, 16 weeks	Text messaging improved remission rate in intervention group (51%) compared to control group (36.1%).

### Technological Aspects

Regarding technological aspects, text messages were sent from a web-based application or a mobile phone (see Multimedia Appendix 1). In most studies, patients were encouraged to send text messages to caregivers for monitoring purposes (25/36, 69%). In only two studies (2/36, 6%), the patients were the only individuals to send text messages. Eleven studies proposed a one-way caregiver to patient text message exchange. In some studies, mobile devices were provided to patients for the study period [40,54]. One study also used an original smartphone application to manage text messaging [39].

## Discussion

Text messaging is an effective means to assess the impact of mHealth interventions in unbiased samples and in a widespread population. Only 32% of the population worldwide own a smartphone [1]. In comparison, ubiquitous access to text messaging in the general population has recently been reached, making it a much more feasible method for managing psychiatric disorders in the broadest population.

We extensively screened all published papers dealing with the use of text messaging in the field of mental health care. Despite recent rapid growth, this innovative approach is still at an early stage. It remains difficult to interpret research findings because of heterogeneous study designs, populations, and medical conditions. Providing a review that could effectively translate findings into best-practice methods remains impossible, as has been described in other reviews concerning mHealth [4]. We aimed to descriptively report the research that has been conducted in the past decade concerning text messaging in mental health care in terms of medical conditions, characteristics of the interventions, and outcomes.

Results from this literature review demonstrate that text messaging as a management tool for mental health has been proposed in many mental health conditions with promising results. Innovative strategies have been suggested, but there is still a lack of evidence regarding their efficacy due to the paucity of consistent RCTs. We found a growing number of initiatives toward incorporation of text messaging in existing mental health care strategies, most of which were pilot studies. The increase in the number of articles over the past decade also indicates growing interest in the topic in peer-reviewed scientific literature. In particular, the number of articles nearly doubled from 2013 to 2014. These results are consistent with the increasing use of text messaging in all fields of health care management. Text messaging is highly adaptable to any health care strategy, given that it does not interfere with pre-existing care procedures. The findings from these text messaging studies may contribute to innovation in other fields of medicine as well.

### mHealth and the Emergence and Expansion of Ecological Momentary Assessment

Although there are still a number of ways in which text messaging can be improved, it is clear that there are many overarching benefits to the use of mHealth techniques in the clinical field. With increasing availability of technology such as mobile phones and smartphones, not only are new forms of clinical intervention possible, but EMA has also arisen as a powerful research tool, especially in younger populations. Self-assessment and EMA have produced promising results when used in patients with mental disorders. EMA extends the concept of self-monitoring to emphasize real-world, real-time data capture. The ability to reach out to patients on a regular basis via text messaging allows for more effective use of EMA since patients can be assessed in their natural environments, rather than in a hospital or counseling setting. This advantage allows for a more accurate and comprehensive reading of patients’ physical and mental status, thus enabling physicians to better treat patients.

However, little is known about the validity provided by the assessment. In our review, researchers often used custom assessment procedures. An interesting approach, proposed by Altman et al (2003), provides patients with a self-assessment method relying on a validated self-assessment scale. This consideration may be of importance given that other medical specialties also intend to implement EMA (eg, in diabetes and asthma management) and may face the same validity issues [5]. Migration of such methods to an automated environment requires prior validation [7].

### Insight Into the Type of Text Messaging Used

Overall, text messaging was used for reminders, information provision, supportive messages, and self-monitoring. Messages were never sent as a substitute for consulting or treatment. In our review, text messaging was proposed to patients suffering from either acute or chronic conditions. Text messaging invariably provided an extension of traditional care strategies, and this extension may occur after discharge [22], in between counseling sessions, or for delivery of a preventive message to an at-risk population [32]. Even when text messaging was used to perform a single assessment, it had a positive effect on clinical outcomes [49]. This finding is an important point that highlights a common aim in mHealth strategies: the strengthening of continuity in existing care. Text messaging in mental health seems to embrace a broad panel of messaging possibilities, breaking away from an approach that would rely solely on reminders [56]. These findings may encourage initiatives in other fields of medicine to assess the impact of text messaging and all of its available features.

### Managing Patient Refusal

We found that text messaging may be readily accepted, even in populations that might normally be reluctant or opposed to treatment. The attitude of patients toward mental health services is crucial to seeking help, determining pathways of care, and subsequent therapeutic commitment and adherence. In fact, mental health care services often have to deal with patient refusal. Text messaging, however, was well accepted by patients suffering from eating disorders (a population that often refuses traditional treatment), as well as suicide attempters who accepted text messaging after refusing hospitalization [57]. Similar challenges can be overcome through the use of messaging in immunization campaigns [58], insulin therapy [59], and cancer management [60]. Text messaging is able to renew the care-giving process and avoids disruptions in the patient-physician relationship. Text messaging may also be an opportunity for patients to retain access to important health information without the potential stigma associated with clinic visits.

### Reduction of Social Isolation and Increased Patient Interactivity

Text messaging is also particularly useful to reduce social isolation. As with other chronic conditions, mental illness is typically associated with increased social isolation, and text messaging may be an important tool to combat this issue [61]. Social isolation can be defined as disengagement from social ties, institutional connections, and even access to care services. Traditionally, interventions that provide social support for patients with disabling conditions rely on caregivers [62] or outpatient nursing programs [63]. The mechanism for maintaining patient participation may be as simple as a weekly message asking, “*How are you?*” [50] or, “*Thank you*” messages [47]. These spontaneous forms of contact allow patients to self-report their problems to caregivers using text messages, or to seek telephone or face-to-face contact. Two-way text messaging may also allow patients to become more involved in treatment, as shown in patients with asthma or diabetes [3,4]. Overall, it is clear that text messaging can provide effective two-way communication and promising support to enhance patient involvement and interactivity, and ultimately to reduce social isolation.

### Limits and Recommendations

In our study, we propose a review of mHealth interventions based on text messaging. Despite the many benefits of SMS text messaging, there are still some limitations to the use of text messages in the clinical realm, which need to be addressed. Since the inception of text messaging, other innovative mHealth strategies have been developed using recent technological advances [64]. Due to the simplicity of its content, text messaging cannot be used as a remote counseling tool, unlike other telemedicine devices [6]. However, even with a few words, a simple message can have an important impact. The content of messages is of particular importance. Some characteristics such as personalization, caring sentiments, and polite text are associated with more successful preventative messages [65]. Most texting applications in mental health share a common characteristic: they tend to enhance connectedness between patient and caregivers. This concept was introduced before the mHealth era. In a pioneer study conducted in the 1970s, Motto proposed to stay in touch with patients discharged after a suicide attempt through regular surface mail for five years [66]. This effect has been described as testimony of the concern that caregivers have for the patient’s situation. Unfortunately, we did not retrieve any studies that addressed the question of message content. Furthermore, automated sending (which was often used in the included studies) was not conducive to basic precautions regarding message content. More concern and better knowledge is required in this area.

Another limitation present throughout the studies was that baseline behaviors with regard to the use of cellular technology varied widely between population subgroups [1,67]. We found only one study that assessed the use of mobile phones before enrolment in the study [37]. Since important variations have been described depending on population subgroups, better description of the population studied could help identify which patients would most likely respond favorably to text messaging. In the general population, the desire to use mobile phones also varies depending on age [68] and gender [69]. A better description of sociodemographic patterns may also be of crucial importance in selecting the right intervention for the right subgroup. Other factors such as level of education seemed to be associated with a better response to preventive messaging [70]. Such idiosyncratic factors may help predict the effects of text messaging. Insight into these factors likely requires better assessment of baseline behaviors.

Several important ethical considerations related to the use of text messaging in mental disorders must also be considered. The matter of participant burden is one such issue. Text messaging entails a non-negligible time commitment on behalf of the participants, and some text messaging programs rely on daily, or sometimes more frequent, prompts. Studies usually last for several days [52] or months, often after discharge or between counseling sessions. Furthermore, recording participants’ daily experiences in a continuous manner is an integral part of EMA. This approach may be significantly more invasive than asking a participant to complete a retrospective questionnaire or answering a question at a traditional interview. The risk of intrusiveness into daily life is real, and yet this issue was not assessed in the articles we reviewed or in other reviews in the field [71]. Receiving a text message may also inconvenience participants who are expected to complete an EMA or read information at a moment’s notice (ie, requests may occur at inopportune times). Caregivers should ascertain that such a burden would not be detrimental to participants’ well-being, particularly when studying individuals who have recently remitted or who are in-episode.

The number of messages can also vary based on the patients’ condition and target behaviors. Some patients may need to receive weekly messages (eg, in suicide management [41]), whereas other patients may need to receive daily messages to remind them to take medication [34] or for monitoring purposes [38]. Pop-Eleches et al [72] tested both daily and weekly messages and found that weekly messages improved anti-retroviral therapy adherence whereas daily messages did not. The frequency of text messages has been described as an important factor for success, as well as failure. In reminding patients to take their medication, weekly messages were more likely to achieve adherence above 95%. Daily messages were likely to be intrusive and cause user fatigue, thereby rendering them ineffective. Further research is necessary to understand the role of timing in the efficacy of text messaging, taking into account the potential intrusiveness of such interventions.

### Conclusion

Overall, it is clear that text messaging has numerous benefits, from extending EMA and increasing patient interactivity to improving mental health in patients with chronic conditions, and encouraging treatment in normally resistant populations. Text messages and mHealth interventions are at a crucial stage in their development, as they present a promising opportunity for innovation in medicine, especially in terms of connectedness between patients and care services. At the same time, the risk of intrusiveness linked to the entry of care services into a patient’s personal space is high. This risk should be more carefully assessed. Nevertheless, text messages allow for inexpensive and instantaneous communication between patients and clinicians, and remains the easiest way to access mHealth applications. All results could be transferred to prompting applications using smartphone technologies. Additionally, early studies suggest that text messaging may be helpful for treatment adherence. Text messaging could also be useful for other aspects of patient self-management, by enhancing social support, encouraging patients to become more proactive in health care, and providing information to enhance health and well-being.

## Appendix

Multimedia Appendix 1 Technical features of text-messaging procedure.

## Abbreviations

EMA: ecological momentary assessment
mHealth: mobile-health
PTSD: post-traumatic stress disorder
RCT: randomized control trial
SMS: short message service
