# Supplementary material for: Fundamentals for Future Mobile-Health (mHealth): A Systematic Review of Mobile Phone and Web-Based Text Messaging in Mental Health
Source: J Med Internet Res. 2016 Jun 10;18(6):e135. doi: 10.2196/jmir.5066 (PMC4920962; doi:10.2196/jmir.5066)
Supplement: Multimedia Appendix 1 [file jmir_v18i6e135_app1.pdf]

Multimedia Appendix 1: Technical features of text-messaging procedure

Authors (year)	Sending procedure	Communication features	Self-monitoring	Content of messages	Frequency, duration of messaging
Suffoletto (2011)	Web-based	Two-way	Custom	Identical	Weekly, 12 weeks
Stoner et al. (2012)	Web-based	One-way	No	Identical	Daily, 8 weeks
Haug et al. (2013)	Web-based	Two-way	Custom	Tailored	Daily to weekly, 12 weeks
Keoleian et al. (2013)	Web-based	Two-way	No	Personalized	Daily, 23 days
Mason et al. (2013)	Web-based	Two-way	Custom	Personalized	3 to 4 messages a day
Rios-Bedoya et al. (2013)	Web-based	Two-way	Text-messaging survey	Standard	Two surveys
Bendsten et al. (2014)	Web-based	Two-way	Custom	Identical	Four days a week, 4 weeks
Lucht et al. (2014)	Web-based	Two-way	Custom	Personalized (nominative)	2 messages per week, 8 weeks
Moore et al. (2014)	Telephone sending	Two-way	Relevant scale	Identical	Daily
Rachel Gonzales et al. (2014)	Web-based	Two-way	Custom	Individualized, tailored	Daily, 12 weeks
Suffoletto et al. (2014)	Web-based	Two-way	Custom	Individualized	Twice a week, 12 weeks
Pijenborg et al. (2010)	Web-based	One-way	No	Personalized according to patient's goals	Depending on patient's wishes, 7 weeks
Depp et al. (2010)	Web-based	Two-way	Custom	Standard initially, based upon patient responses	6 days a week, 12 weeks
Granholm et al. (2012)	Web-based,	Two-way	Multiple-choice protocol	Personalized based on patient response	6 days a week, 12 weeks
Maritta Välimäki et al. (2012)	Semi-automated web-based	One-way	None	Custom	Custom frequency, 12 months
Montes et al. (2012)	Web-based	One-way	None	Standard	Daily reminders, 3 months
Ainsworth et al. (2013)	Web-based	Two-way	Custom	Standard	Daily, 3 weeks
Bebee et al. (2014)	Web-based	Two-way	Custom multiple choice	Daily text messages for EMA	Daily, 3 months
Ben-Zeev et al. (2014)	Telephone sending	Two-way	Custom	Both identical and personalized	3 messages a day for 12 weeks
Van den Berg et al. (2011)	Web-based	Two-way	Custom	Standard	Weekly, 6 months

Bopp et al. (2010)	Web-based	Two-way	Relevant scale	Altman Self-Rating Mania Scale	Weekly, 36 weeks
Agyapong (2013)	Web-based	One-way	No	Standard among 10 possible messages	Twice daily, 3 months
Agyapong (2012)	Web-based	One-way	No	Identical	Daily, 3 month
Aguilera et al. (2011)	Manual and automated sending	One-way	None	Standard and custom	Daily
Aguilera et al. (2014)	Manual and automated	One-way	None	Standard and custom	Daily
Chandra et al. (2014).	Telephone sending	One-way	None	Identical	Daily, one month
Kunigiri et al. (2014).	Telephone sending	One-way	None	Standard	Evening before appointment
Branson CE et al. (2013).	Web-based	One-way	None	Standard	Two days before appointment
Price et al. (2014).	Web-based	One-way	Custom	Standard	Daily, 15 days
Furber et al. (2014)	Web-based	One-way	Custom	Customized	Schedule decided on day-to-day basis
Chen et al. (2011)	Telephone sending	Two-way	None	Standard	Once a week, 4 weeks
Berrouigut et al. (2014)	Web-based	One-way	None	Personalized	After discharge: 48 hours, then weekly for one month
Berrouiguet et al. (2014)	Web-based	One-way	None	Personalized	After discharge: 48 hours, then monthly for 5 months (total 6 months)
Robinson et al. (2006)	Web-based	Two-way	Custom	Customized messages	Weekly, Six months
Shapiro et al. (2010)	Web-based	Two-way	Custom Likert-based assessment	Daily feedback message among 100	Daily, 12 weeks
Lucht et al. (2014)	Web-based	Two-way	Custom	Tailored messages	Weekly, 16 week
